# Thuja in Ovaritis of the Left Side

**Published:** 1889-08

**Authors:** B. Simmons

**Affiliations:** Sydney, N. S. W.


### THUJA IN OVARITIS OF THE LEFT SIDE.

During the early years of my medical practice I was extremely ignorant of the materia medica, a condition which almost continual ill-health did not improve, as the inductive method of Hahnemann is no easy matter for invalids. In those days I obtained the assistance of that great expert in the materia medica, Dr. David Wilson, of London, of whose kindness, courtesy, and skillful help it would be impossible for me to speak in sufficiently high terms. About this time I was consulted by a lady who had recently been confined, and was suffering from severe pain in the *left ovarian region, extending down the thigh;* any attempt at *exercise*, such as *walking*, greatly aggravated the suffering. Dr. Wilson advised Thuj.$^{200}$, three times a day. A prompt cure resulted. Since that time I have met with a large number of cases presenting similar symptoms. Thuj. has in most cases effected a cure. I look upon it as the most frequently indicated remedy for ovarian mischief on the left side; its only rival seems to be Lil. tig.—the differentiation is, however, easy, for, in the latter remedy, *bearing-down* pain is prominent, and is *relieved by external pressure*, and when this is associated with an early morning diarrhœa, driving the patient hurriedly out of bed (like Sul.), the preference must be given to Lilium. There are, of course, many other medicines for left ovarian pain, such as Lach., Argent., Brom., Phos., Coloc., Ust., Graph., etc.; still, for the sake of the junior members of the profession, I would call attention to Thuj. in this distressing malady. Dr. Guernsey, in his *Obstetrics*, gives due prominence to it, but in spite of this one sees very few reported cases illustrating its marvelous curative power. In the March number of THE HOMŒOPATHIC PHYSICIAN, 1885, there is an article on "*Psora and Syphilis*," by Dr. Woolf. The information therein contained is of a most valuable character, suggesting the study of remedies for sycotic and syphilitic cases—Thuj., of course, occupying the first position, where gonorrhœa or vaccination may be regarded as the probable cause. In the evil effects of the latter, the study of Sil. is suggested by our immortal Hering, and in many cases it would be found to be the true simillimum. Some years ago, when traveling through North Wales, I met with a young man about twenty-seven years of age, who was suffering from the ill effects of vaccination. Up to the time of the performance of the operation, he had been a strong and vigorous man, but since that time his health had become so broken that he had not had a single day's health, and after having taken much physic, prescribed by leading physicians in London, without any good effect, was traveling in the hope of shaking off his miseries. Having no Repertory with me, I prescribed one globule of Sil.$^{30}$, night and morning, being solely guided by the recommendation of Hering. I saw or heard nothing of my new acquaintance until six months afterward, when he wrote me, stating that for the first month he received no benefit, but that after that time his health and strength had gradually returned, and that now he was perfectly well, and had for some time been discharging his duties as a clergyman. I regret that I took no record of the symptoms then present, but the result is, at any rate, a useful observation in showing the good effects of Sil. in the sycotic condition developed by vaccination.

In the year 1865, during an epidemic of small-pox, I vaccinated several children, including one of my own, with lymph taken from an apparently healthy child, chosen specially for that purpose. In one case dysentery followed, but in nearly all evidences of impure lymph manifested themselves. My son developed small boils over the body, with flat yellow crusts; he also fell away in health, but after a time seemed to have recovered. Some ten years after this he took cold by getting his feet wet in the snow, and became gradually very deaf. Several remedies were given to him, under the direction of Dr. Wilson, but, as the symptoms were somewhat vague, his condition remained unchanged. At length he became suddenly worse, feeling ill and being feverish, and after being a short time in bed in the evening, was attacked with earache of a violent, tearing, shooting character. During the paroxysms of pain he *frequently rose to urinate*, which seemed a peculiar concomitant. Dr. Wilson now advised Thuj.$^{200}$ every four hours, a prescription which was followed by a rapid and permanent cure. The only other symptoms present in this case were a stopped-up feeling in the ears, which sometimes felt painful during the act of coughing. On page 974 of Jahr's *New Manual*, under "Thuj.," we find, "In the evening when in bed he experiences a terrible hammering and tearing in the ear until after midnight, accompanied with micturition every half-hour, and coldness of the legs up to the knees." Of such cures as these a physician may well be proud, but they can only be performed by a strict adherence to the principles of Hahnemann.

B. Simmons, M. D.

Sydney, N. S. W.

